# Cloning, Expression and Characterization of UDP-N-Acetylglucosamine Enolpyruvyl Transferase (MurA) from *Wolbachia* Endosymbiont of Human Lymphatic Filarial Parasite *Brugia malayi*

**DOI:** 10.1371/journal.pone.0099884

**Published:** 2014-06-18

**Authors:** Mohd Shahab, Meenakshi Verma, Manisha Pathak, Kalyan Mitra, Shailja Misra-Bhattacharya

**Affiliations:** 1 Academy of Scientific and Innovative Research (AcSIR), Anusandhan Bhawan, New Delhi, India; 2 Division of Parasitology, CSIR-Central Drug Research Institute, Lucknow, Uttar Pradesh, India; 3 Electron Microscopy, CSIR-Central Drug Research Institute, Lucknow, Uttar Pradesh, India; New England Biolabs, United States of America

## Abstract

*Wolbachia,* an endosymbiont of filarial nematode, is considered a promising target for treatment of lymphatic filariasis. Although functional characterization of the *Wolbachia* peptidoglycan assembly has not been fully explored, the *Wolbachia* genome provides evidence for coding all of the genes involved in lipid II biosynthesis, a part of peptidoglycan biosynthesis pathway. UDP-N-acetylglucosamine enolpyruvyl transferase (MurA) is one of the lipid II biosynthesis pathway enzymes and it has inevitably been recognized as an antibiotic target. In view of the vital role of MurA in bacterial viability and survival, MurA ortholog from *Wolbachia* endosymbiont of *Brugia malayi* (*w*Bm-MurA) was cloned, expressed and purified for further molecular characterization. The enzyme kinetics and inhibition studies were undertaken using fosfomycin. *w*Bm-MurA was found to be expressed in all the major life stages of *B. malayi* and was immunolocalized in *Wolbachia* within the microfilariae and female adults by the confocal microscopy. Sequence analysis suggests that the amino acids crucial for enzymatic activity are conserved. The purified *w*Bm-MurA was shown to possess the EPSP synthase (3-phosphoshikimate 1-carboxyvinyltransferase) like activity at a broad pH range with optimal activity at pH 7.5 and 37°C temperature. The apparent affinity constant (*K*_m_) for the substrate UDP-N-acetylglucosamine was found to be 0.03149 mM and for phosphoenolpyruvate 0.009198 mM. The relative enzymatic activity was inhibited ∼2 fold in presence of fosfomycin. Superimposition of the *w*Bm-MurA homology model with the structural model of *Haemophilus influenzae* (*Hi-*MurA) suggests binding of fosfomycin at the same active site. The findings suggest *w*Bm-MurA to be a putative antifilarial drug target for screening of novel compounds.

## Introduction

*Wolbachia* are the maternally inherited intracellular gram negative alphaproteobacteria widely spread among arthropods and filarial nematodes exhibiting a diverse range of associations with their host. In filarial nematodes, they exhibit vertical transmission via oocytes that has promoted evolutionary adaptation and a mutualistic relationship.

Lymphatic filariasis (LF), the cause of long-term disability in tropical and sub-tropical countries is caused by the filarial nematodes, *Wuchereria bancrofti* and *Brugia* species. Over 120 million people are currently infected and one third of these develop major morbidity world-wide [1]. Currently used antifilarial drugs interrupt transmission of infection by principally killing the larval stage called microfilariae (mf) without much effect on the adult parasites. Since the adult filarial worms can survive up to decade in the vertebrate host, repeated annual treatments are recommended for several years to bring the mf density to a very low level that will not transmit infection. *Wolbachia* is obligatory for most species of filarial nematodes as evidenced by the killing of *Wolbachia* following tetracycline and doxycycline treatment that impairs the development and fecundity of worms [2]–[5]. However, the antibiotics require long course of treatment and are not recommended for use in the young children and pregnant women [6]. In absence of an adulticidal drug together with the threat of drug resistance to mainstay filaricides [7], [8], identification and characterization of novel antifilarial drug targets and discovery of novel classes of compounds with different mode of action is urgently required. *Wolbachia* bears extremely low number of predicted genes (∼806) as compared to other bacteria [9] which include several unique potential targets [10]. Investigations on a few proteins/enzyme pathways of *Wolbachia* have recently been undertaken such as *Wolbachia* surface protein (WSP), heat shock protein 60 (HSP60), independent phosphoglycerate mutase (*i*PGM), pyruvate phosphate dikinase (PPDK), enzymes regulating heme, lipid II and lipoprotein biosynthesis [11], NAD-dependent DNA ligase (*w*Bm-LigA) [12] and transcription factor [13]. Specific inhibitors of few *Wolbachia* enzymes have been investigated recently. The benzimidazoles have been shown to inhibit heme biosynthesis pathway [14], acyldepsipeptides inhibit Clp peptidase [15] and heteroaryl compounds target rsmD-like rRNA methyltransferase [16]. These inhibitors also exhibited antifilarial activity reassuring *Wolbachia* as a promising antifilarial drug target.

**Figure 1:**
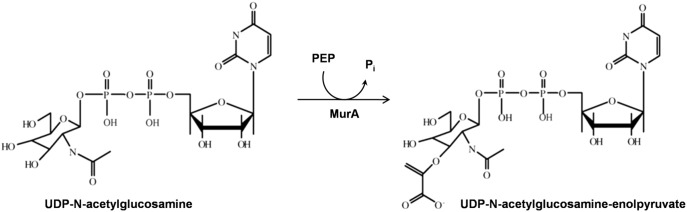
The first cytoplasmic step of the peptidoglycan biosynthesis. MurA catalyses the formation of UDP-N-acetylglucosamine-enolpyruvyl from phosphoenolpyruvate (PEP) and UDP-N-acetylglucosamine (UDPAG).

Peptidoglycan (PG), an essential component of the cell wall provides structural integrity to bacteria against internal osmotic pressure [17]. The enzymes linked to PG synthesis remain conserved among the bacterial species. These have no mammalian counterpart and therefore present an attractive drug target. The annotated genome of *Wolbachia* reveals the presence of genes required for lipid ΙΙ precursors for PG biosynthesis including UDP-N-acetylglucosamine enolpyruvyl transferase (MurA) [9], [18]. However, the role of lipid ΙΙ in *Wolbachia* remains unclear since other genes involved in PG synthesis such as those responsible for polymerization of glycans are absent. It is well known that MurA catalyzes the first committed step in the cell wall biosynthesis of bacteria and transfers an enolpyruvyl group from phosphoenolpyruvate (PEP) to UDP-N-acetylglucosamine (UDPAG) to form UDP-N-acetylglucosamine enolpyruvate [19] which is a precursor to UDP-N-acetylmuramate, a requisite building block of bacterial cell wall (Figure 1) [20]. However, it has also been shown that inhibition of lipid II synthesis brings about a detrimental effect on *Wolbachia* within the insect cell lines [18]. The deletion/inactivation of MurA gene of *Escherichia coli* [21], *Streptococcus pneumoniae* [22], and *Staphylococcus. aureus* [23] has been shown to cause their death and this has been investigated extensively.

In view of high homology of the protein encoded by UDP-N-acetylglucosamine enolpyruvyl transferase gene in all the related bacteria, it is quite likely that *w*Bm-MurA may also perform similar function in *B. malayi* endosymbiont. The structure prediction studies in the current study establish that the key amino acids required for MurA enzymatic activity are conserved in *w*Bm-MurA. In addition, fosfomycin brought about an irreversible inhibition in *w*Bm-MurA activity as also reported earlier in other bacteria [24]. The current investigation deals with the cloning, expression, purification and characterization of *B. malayi Wolbachia* MurA.

## Materials and Methods

### Animals, Parasites, Infection

Rodent host *Mastomys coucha* (GRA ‘Giessen’ strain) were infected with *B. malayi* (sub-periodic strain) by subcutaneous inoculation of 100 third stage infective larvae (L3) isolated from laboratory bred mosquito vector *Aedes aegypti* using Baermann technique [25], [26]. Jirds (*Mastomys unguiculatus*) were infected with 150–200 *B. malayi* infective larvae by the intraperitoneal route [27]. Adult parasites and microfilariae (mf) were recovered from the peritoneal cavity of jird infected 15–20 weeks earlier. Adult parasites were made free of host tissues under the dissecting microscope and mf were pelleted by passing the peritoneal wash through a 5.0-µm membrane filter in sterile phosphate buffer saline (PBS-pH 7.2). Euthanization of animals was done by injecting overdose of Intraval Sodium (100 mg/kg). Efforts were made to minimize suffering and reduce the number of animals used. All studies involving animals are reported in accordance with the ARRIVE guidelines for reporting experiments involving animals [28].

### Ethics Statement

The animals used in the study were housed in climatically controlled pathogen free rooms at National Animal Laboratory Centre, CSIR - Central Drug Research Institute, Lucknow, India and fed with standard pellet diet with water *ad libitum*. This study was approved by the Animal Ethics Committee of CSIR - Central Drug Research Institute, Lucknow, India constituted under the rules and guidelines of the Committee for the Purpose of Control and Supervision of Animals (CPCSEA), Government of India (1998). The study bears approval nos. IAEC/2011/120 & IAEC/2011/145.

### Genomic DNA Isolation, Amplification and Cloning of *w*Bm-MurA Gene

Adult worms were harvested from the euthanized jirds. Worms were washed repeatedly in PBS and genomic DNA was isolated following manufacturer’s protocol (PureLink Genomic DNA mini kit-Invitrogen). The genomic DNA from adult also contains genomic DNA of *Wolbachia.* 1278 bp *w*Bm-MurA gene (KEGG - *w*Bm0740) was amplified from the extracted genomic DNA using forward sense primer - 5′-GGATCCATGCATAAAATATTAGTAAGGAGTAAC-3′ and reverse antisense primer - 5′-CTCGAGTCAAGGAATAGAGATATCGGCCC-3′ containing restriction sites *Bam*HI and *Xho*I (underlined), respectively. The amplification was carried out by mixing 1 µM of each primer, 200 µM of each deoxynucleoside triphosphate (dNTPs), 0.5 unit *Taq* DNA polymerase, 1x PCR buffer, and 1.5 µM MgCl_2_ (all from Fermentas) in a thermocycler (Bio-Rad) under conditions at initial denaturation at 94°C/4 min, 29 cycles at 94°C/45 sec, 48°C/45 sec, 72°C/1.30 min and 1 cycle at 72°C/20 min. The amplified PCR product was electrophoresed in agarose gel and eluted by gel extraction kit (PureLink Gel Extraction kit, Invitrogen). Eluted product (∼1278 bp) was sub-cloned into pTZ57R/T (T/A) cloning vector (Fermentas) and transformed into competent *E. coli* DH5α cells. The transformants were screened for the presence of recombinant plasmids with the desired insert by gene specific PCR under similar conditions as mentioned above. Cloning was performed at *Bam*HI and *Xho*I site in bacterial expression vector pET28a (Novagen) and the plasmid from positive clones was sequenced to confirm the insert.

### *w*Bm-MurA Gene Expression in Various Stages of *B. malayi*

Different life stages of *B. malayi,* viz. adult, mf and L3 were recovered as detailed above. RNA was extracted using the TRIzol reagent (Invitrogen) and quantified with a GeneQuant apparatus (Bio-Rad). After treatment with DNase I to remove genomic DNA contamination, 3 µg of total RNA from each life stage was used for cDNA synthesis using a first-strand cDNA synthesis kit (Sigma-Aldrich, USA). The target gene was amplified using cDNAs applying conditions as mentioned above. For negative controls, PCR was performed with total RNA in absence of reverse transcriptase, in order to rule out any possibility of DNA contamination in the total RNA samples.

### Expression, Purification and Western Blot

The expression of recombinant *w*Bm-MurA was checked in bacterial cells by transforming the recombinant construct in *E. coli* - Rosetta(DE3)pLysS strain (Novagen). The transformed cells were inoculated into 5 ml Luria-Bertani medium and allowed to grow at 37°C in a shaker at 220 rpm. Cultures in logarithmic phase (OD_600_∼0.5–0.6) were induced for 3 h with different concentration of isopropyl-β-D-thiogalatopyranoside (IPTG) at 37°C. The over-expression of the recombinant (r) *w*Bm-MurA was analyzed by 10% sodium dodecyl sulfate - polyacrylamide gel electrophoresis (SDS-PAGE) after Coomassie brilliant blue R-250 (Sigma-Aldrich) staining.

For purification, 300 ml Luria-Bertani medium containing 100 µg/ml-chloramphenicol and 50-µg/ml kanamycin were inoculated with freshly transformed pET28a recombinant construct and grown at 37°C/220 rpm to an OD of ∼0.6. For expressing the recombinant protein in soluble form, the culture was further grown at 24°C for 20 min., induced by the addition of 0.2 mM IPTG and further grown for 22 h at 24°C/130 rpm. The recombinant *w*Bm-MurA was purified by affinity chromatography using Ni^2+^ chelating resin which binds to (His)-6-tag fusion peptide derived from the pET28a vector. Harvested cell pellet was re-suspended in 15 ml of chilled lysis buffer (20 mM Tris-pH 7.4, 300 mM NaCl, 1 mM EDTA, 10% Glycerol, 1% Triton X-100, 0.5 mM phenyl-methanesulfonyl fluoride [PMSF], 5 mM β-mercaptoethanol) and incubated for 45 min on ice with 1mg/ml of lysozyme (Sigma-Aldrich). The suspension was disrupted by sonication (20 cycles; 10 sec pulse at 20% amplitude with 30 sec interval after each pulse) on ice, and pelleted at 12,500 rpm for 30 min. The supernatant was incubated at 4°C for 1 h with 3 ml Ni-NTA resin (Qiagen,) in a column pre-equilibrated with lysis buffer. The column was subsequently washed with lysis buffer only and then with wash buffer (20 mM Tris-pH 7.4, 300 mM NaCl, 1 mM EDTA, 150 µM PMSF) containing different concentrations of imidazole (20, 40 and 60 mM). The purified recombinant protein was eluted with wash buffer containing 300 mM imidazole. All the washing, elution and dialysis step were performed at 4°C. For purity check, 100 µl from each eluted fraction was mixed with an equal volume of 2x sample buffer (10 mg/ml Bromophenol Blue, 4.4% SDS, 0.5 M Tris-Cl, 300 mM β-mercaptoethanol) and analyzed on 10% SDS-PAGE along with un-induced sample fraction. The protein was dialyzed against Buffer A (20 mM Tris-Cl-pH 7.4, 250 mM NaCl, 50 mM imidazole) and subsequently against Buffer B (20 mM Tris-Cl-pH 7.4, 250 mM NaCl). Concentration of the eluted fractions was estimated by the Bradford method using Bovine serum albumin (BSA) as standard [29]. The resolved purified recombinant protein was transferred to a nitrocellulose membrane in a mini-blot transfer assembly (Bio-Rad). The membrane was blocked in 3% skimmed milk for 2 h at room temperature (RT). After blocking, the membrane was incubated at RT with mouse anti-His antibody (Novagen, USA) at 1∶2000 dilution. The membrane was washed thrice with PBS containing 0.5% Tween 20 and then incubated with goat anti-mouse IgG-HRP conjugate (Sigma, USA) at a dilution of 1∶10,000 for 2 h at RT. The blot was developed with 3,3′-diaminobenzidine tetra hydrochloride (DAB) and H_2_O*_2_* (Sigma-Aldrich).

### Raising Polyclonal Antibodies Against *w*Bm-MurA

For the generation of polyclonal antibodies to *w*Bm-MurA, 20 µg of *w*Bm-MurA recombinant protein mixed with Fruend’s complete adjuvant (FCA, Sigma, USA) in 100 µl volume was administered subcutaneously in six eight-week old BALB/c mice. Further two booster doses of same amount of protein were given with Fruend’s incomplete adjuvant (FIA, Sigma, USA) on the day 15 and 21 post first immunization. Anti-*w*Bm-MurA serum was collected from the blood collected on day 30 post first immunization.

### Stage Specific Endogenous Presence of *w*Bm-MurA Enzyme

To observe the presence of *w*Bm-MurA protein in adults, L3 and mf of *B. malayi,* the crude extracts from each life stage were resolved on 10% SDS-PAGE prior to the Western blot. The target protein was recognized with polyclonal antibody to *w*Bm-MurA. Crude extract were prepared by homogenization and sonication of 20 adult worms (female and male), ∼4000 L3 and ∼5000 mf in 400 µl PBS, each containing protease inhibitor cocktail (Sigma, USA) followed by the centrifugation at 12,000×g for 30 min. Samples for SDS-PAGE were prepared by mixing the supernatants with an equal volume of 2x sample buffer and heated for 5 min at 100°C. The separated protein fractions from SDS-PAGE were then transferred to the nitrocellulose membrane and the remaining steps were same as appended above for His-fused *w*Bm-MurA Western blotting except that the anti-*w*Bm-MurA mouse serum was used as a primary antibody (1∶5000 dilutions). The purified recombinant *w*Bm-MurA served as a positive control.

### Immunolocalization of *w*Bm-MurA in *B. malayi* by Confocal Microscopy

For observing the *w*Bm-MurA distribution in the parasites, confocal microscopy was undertaken. The adult female worm and mf were fixed overnight in 4% paraformaldehyde in M9 buffer (22 mM KH_2_PO_4_, 42 mM Na_2_HPO_4_, 86 mM NaCl, and 1 mM MgSO_4_. 7H2O; pH 7.2) at 4°C and further processed, as described earlier [30]. Anti-*w*Bm-MurA polyclonal antibody was used as primary antibody (1∶500) while FITC (Fluorescein isothiocyanate; Sigma) conjugated IgG (1∶200) was used as the secondary antibody, both diluted in the M9 buffer with 0.5% BSA respectively. After every step washing was done four times with the M9 buffer containing 0.05% Tween-20. The worms and mf were incubated with 4′,6′- diamidino-2-phenylindole (DAPI, 100 ngml^−1^; Sigma) for 5 min for the DNA staining and parasites were mounted on glass slide in 90% glycerol and 10% p-phenyenediamine (Sigma, USA) in PBS. Slides were analyzed under a Carl Zeiss LSM 510 META (Zeiss, Jena, Germany) confocal laser scanning microscope equipped with 405nm diode, Argon multiline (458, 477, 488, 514nm), 561 nm DPSS and HeNe 633 nm lasers. Plan-apochromat 63X/1.4 NA oil DIC objective and Plan-apochromat 40X/0.95 NA DIC objective along with appropriate excitation and emission filter sets were used for imaging. 488 nm and 405 nm laser lines were used for excitation of FITC and DAPI respectively. As a negative control, the same procedure was executed after treating parasites with BALB/c preimmune serum.

### Sequence Analysis and Phylogeny

BLASTP (http://blast.ncbi.nlm.nih.gov/Blast.cgi) search was made with the *w*Bm-MurA as the query sequence for identifying similar domain sequences. Domain analysis was done on SMART server [31], [32]. To identify the conserved regions, sequence alignments of MurA were generated with ClustalW2 [33]. Phylogenetic tree was constructed by neighbor-joining methods using the programs NEIGHBOR and PROTDIST of the PHYLIP package v3.6 [34]. The programs SEQBOOT and CONSENSE from the same package were used to estimate the confidence limits of branching points from 1000 bootstrap replication. For this, twenty four homologous protein sequences of MurA were retrieved from NCBI databases using BLASTP and aligned using ClustalW software and these are; *Deinococcus radiodurans* (NP_294847.1), *Thermotoga maritima* (NP_227924.1), *Streptococcus pneumoniae* (YP_006700754.1), *Enterococcus faecalis* (WP_002367154.1), *Bacillus subtilis* (NP_391557.1), *Chlamydia trachomatis* (YP_008443514.1), *Porphyromonas gingivalis* (WP_021679280.1), *Treponema pallidum* (NP_218469.1), *Borrelia burgdorferi* (WP_010254843.1), *Mycobacterium tuberculosis* (NP_215831.1), *Synechocystis sp.* (NP_442129.1), *Helicobacter pylori* (WP_000346463.1), *Neisseria meningitidis* (WP_002245281.1), *Vibrio cholerae* (WP_000410583.1), *Escherichia coli* (WP_001545447.1), *Haemophilus influenzae* (YP_005829704.1), *Pseudomonas aeruginosa* (WP_009875898.1), *Acinetobacter calcoaceticus* (WP_005049013.1), *Wolbachia* endosymbiont of *Onchocerca ochengi* (YP_006555880.1), *Wolbachia* endosymbiont of *Drosophila melanogaster* (NP_966909.1), *Wolbachia* endosymbiont strain TRS of *Brugia malayi* (YP_198570.1), *Wolbachia* endosymbiont of *Culex quinquefasciatus* (WP_007302175.1), *Rickettsia prowazekii* (NP_220950.1) and *Aquifex aeolicus* (NP_213879.1). Viewing and re-annotation were done on FigTree v1.4 (http://tree.bio.ed.ac.uk/software/figtree).

### *w*Bm-MurA Activity Assay

In *E. coli*, MurA catalyzed the enzymatic reaction with the release of free inorganic phosphate (P_i_) and this method was used for the enzymatic assays. Reaction was performed in a 96 well plate at 37°C for 10 min in a final volume of 50 µl reaction mixture containing 50 mM Tris-HCl-pH 7.5, 10 mM KCl, 1 mM dithiothreitol (DTT), 10% (v/v) glycerol, 0.6 mM UDPAG (Sigma), and 3 µg of pre-dialyzed *w*Bm-MurA as described earlier [24], [35]. 1 mM PEP (Sigma) was added to initiate the reaction. The amount of P_i_ released due to PEP cleavage was quantified by malachite green assay kit following manufacturer’s protocol (Cayman Chemical Company, USA). Absorbance was read at 620 nm and compared with the standard phosphate solutions. Standard curve was plotted using phosphate standards and regression analysis was performed to estimate the liberated P_i_. For controls, the reaction conditions remained same as mentioned above however *w*Bm-MurA was either inactivated by boiling or it was not added to the reaction mixture. The pH and temperature conditions for optimal enzymatic reaction were assessed at different pH (4–10) and temperature (10–80°C) ranges. For examining the effects of different ions, reactions were performed by replacing KCl with 10 mM of NaCl, NH_4_Cl, CaCl_2_, MgCl_2_, MnCl_2_, CuCl_2_, NiCl_2_, CoCl_2_ and FeSO_4._

### Determination of Kinetic Constants

To determine the kinetic constants at optimum pH and temperature, the above enzymatic assay was performed in presence of various concentration of one substrate with a fixed concentration of another one. Varying concentration of UDPAG (0.0 to 6.4 mM) in presence of 1 mM PEP or varying concentration of PEP (0.0 to 6.4 mM) in presence of 5 mM UDPAG was used. Samples were analyzed at 620 nm for the P_i_ activity over a period of 10 minutes. The average activity (nmol P_i_/min) was determined and eventually *K*_m_ and V*_max_* values were estimated by fitting the curve through non-linear regression by plotting Michaelis-Menton graph [36].

### Inhibition of *w*Bm-MurA Activity by Fosfomycin

Fosfomycin inhibits MurA by making a covalent adduct with the active residue. To determine its inhibitory effects on enzymatic activity, different concentrations (1 to 50 mM) of fosfomycin were pre-incubated with the assay mixture for 15 min and the enzymatic reactions were initiated by addition of 5 mM of PEP. Fosfomycin acts as a PEP analogue, therefore to examine the competitive profile with the PEP, another assay was performed in which the reaction was initiated with different concentrations (1–50 mM) of PEP and a fixed concentration (30 mM) of fosfomycin. The activity was analyzed for both the assays as mentioned above except for the longer incubation time of 1 h due to expected low release of inorganic phosphate. Concentrations of all the other substrates were same as in the activity assay reaction mixture.

### Homology Modeling

For examining the perseverance of the active sites and the probable interactions with fosfomycin, a homology model of the *w*Bm-MurA was generated using Phyre^2^ server - Imperial College, London [37], which has different component suite for efficient modeling (multi-template modeling by Poing 1.0, template detection by HHpred 1.51 and disorder prediction by Psi-pred 2.5). The generated model was further refined through ModRefiner server [38]. For evaluation and validation of the model, Ramachandran Plot was generated from PROCHECK [39]. Knowledge-based energy curve for calculating z-score was done by using ProSA-web [40]. Secondary structure analysis was carried out on PDBsum server [41]. All the visualizations were performed on PyMOL (The Pymol Molecular Graphics System, Schrödinger, LLC).

### Statistical Analysis

All the measurements were performed in triplicate and repeated thrice to correct the trial errors. Data were analyzed with the help of statistical software GraphPad Prism (version 6.01).

## Results

### Sequence and Phylogenetic Analysis

**Figure 2:**
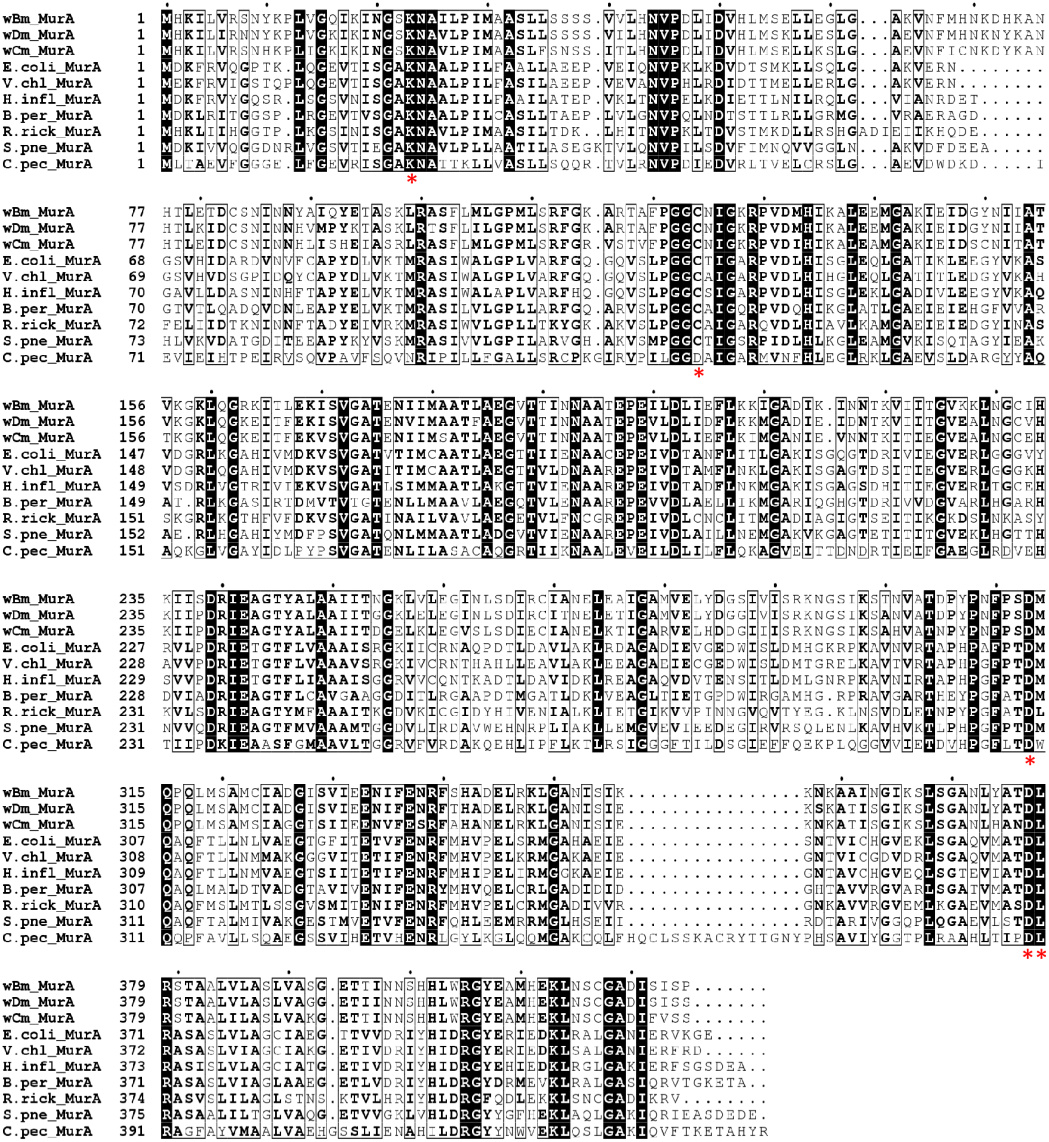
ClustalW analysis of the amino acid sequences of MurAs. Sequences from various organisms were aligned (NCBI reference numbers are given in parenthesis). *Wolbachia* endosymbiont of *B. malayi* - wBm_MurA (YP_198570.1), *Drosophila melanogaster* - wDm_MurA (NP_966909), and *Culex molestus* - wCm_MurA (CDH88571); *E. coli*_MurA (WP_023568349), *Vibrio cholerae -* V.chl_MurA (WP_001887759), *Haemophilius influenzae* - H.infl_MurA (YP_005827986), *Bordetella pertusis -* B.per_MurA (WP_014906110), *Rickettsia rickettsii* - R.rick_MurA (WP_012151046), *Streptococcus pneumoniae -* S.pne_MurA (WP_023396463) and *Chlamydia pecurum* - C.pec_MurA (YP_008583337). Conserved motifs are marked and boxed, white letters with black background indicate identical amino acid positions/sequence from various organisms, red star shows the residues involved in the ligand interactions during catalysis. The alignment figure was generated using the ESPript 3.0 server (http://espript.ibcp.fr/ESPript/cgi-bin/ESPript.cgi).

**Figure 3:**
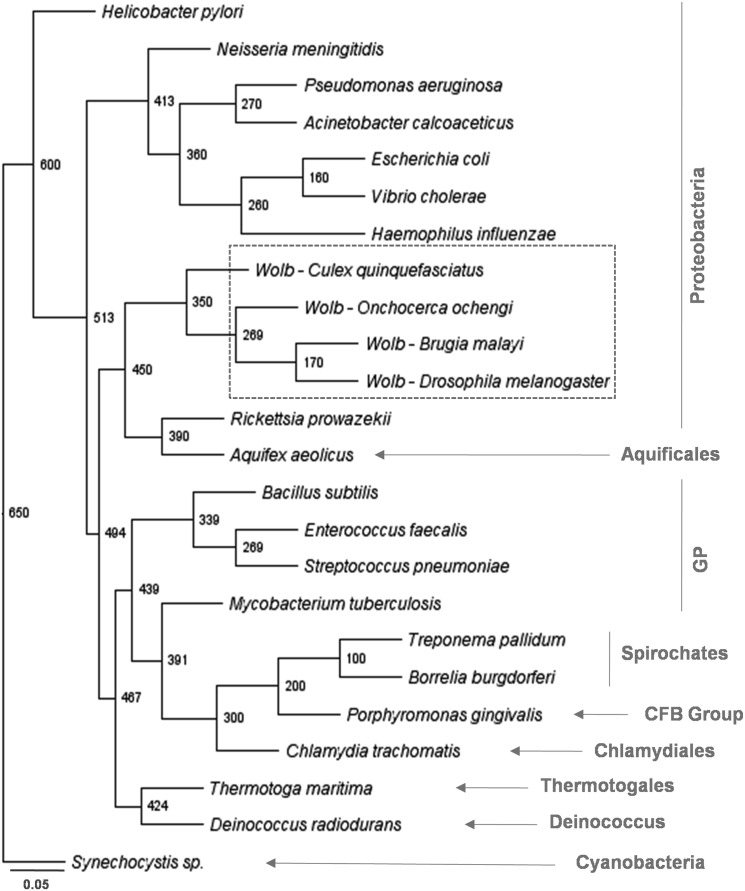
Phylogenetic tree showing divergence of MurA. Tree was constructed by NJ method as implemented by the PHYLIP 3.6 package using the input sequences with 1,000 bootstrap replicates. The scale bar represents 0.05 expected amino acid replacement per site as estimated by the program PRODIST of the same package. Number at the nodes represents the age constraint to mean path lengths. Major groups of bacteria are included with the abbreviation given to gram-positive bacteria (GP), *Cytophaga-Flexibacter-Bacteroids* (CFB) and intracellular *Wolbachia* (*Wolb*). Graphical version of the tree was drawn on FigTree program.

Database search revealed that MurA gene exists in nearly all the bacterial species. *w*Bm-MurA shares high degree of similarity with MurA homologues of other *Wolbachia* species (Figure 2). It exhibits 88.94 and 80.71% homology with MurA of *Wolbachia* of *Drosophila melanogaster* (NP_966909) and *Culex molestus* (CDH88571) respectively. Varying degree of similarity was observed with others bacterial species such as, 41.05% with *Escherichia coli* (WP_023568349), 42.24% with *Vibrio cholerae* (WP_001887759), 40.57% with *Haemophilius influenzae* (YP_005827986), 41.23% with *Bordetella pertusis* (WP_014906110), 43.68% with *Rickettsia rickettsii* (WP_012151046), 43.06% with *Streptococcus pneumoniae* (WP_023396463) and 35.53% with *Chlamydia pecurum* (YP_008583337). In addition, the residues involved in ligand interactions in MurA of *E. coli* (Cys115, Asp305, Lys22, Arg120, Asp369 and Leu370) [22], [42] are conserved among *Wolbachia* of above hosts and also in above bacterial species except *C. pecurum* where the residue complementary to Cys115 of *E. coli* is aspartate. It should be noted that MurA of *C. pecurum* with aspartate substitution is resistant to fosfomycin [43]. Phylogenetic analysis of *w*Bm-MurA via neighbor-joining method displays strong similarity with MurA of other *Wolbachia* endosymbionts and it formed a distinct clade as a characteristic of α-proteobacteria apart from the MurA of the other proteobacteria. Gram positive bacterial species have two copies of MurA in contrast to gram negative bacteria (including *Wolbachia*) which contain only one copy [21]. Phylogenetic analysis suggests that two genes of MurA have possibly evolved by duplication of one gene, or from an even more ancient source as suggested by BLAST homology searches [22]. Thus the phylogram (Figure 3) clearly suggests that MurA gene is evolutionary conserved and may have followed a different path during the time course of evolution. Analysis of the *w*Bm-MurA domain architecture revealed the predicted EPSP synthase (3-phosphoshikimate 1-carboxyvinyltransferase) as the main domain (amino acid, 8 to 414).

### *w*Bm-MurA was Cloned, Expressed and Purified

**Figure 4:**
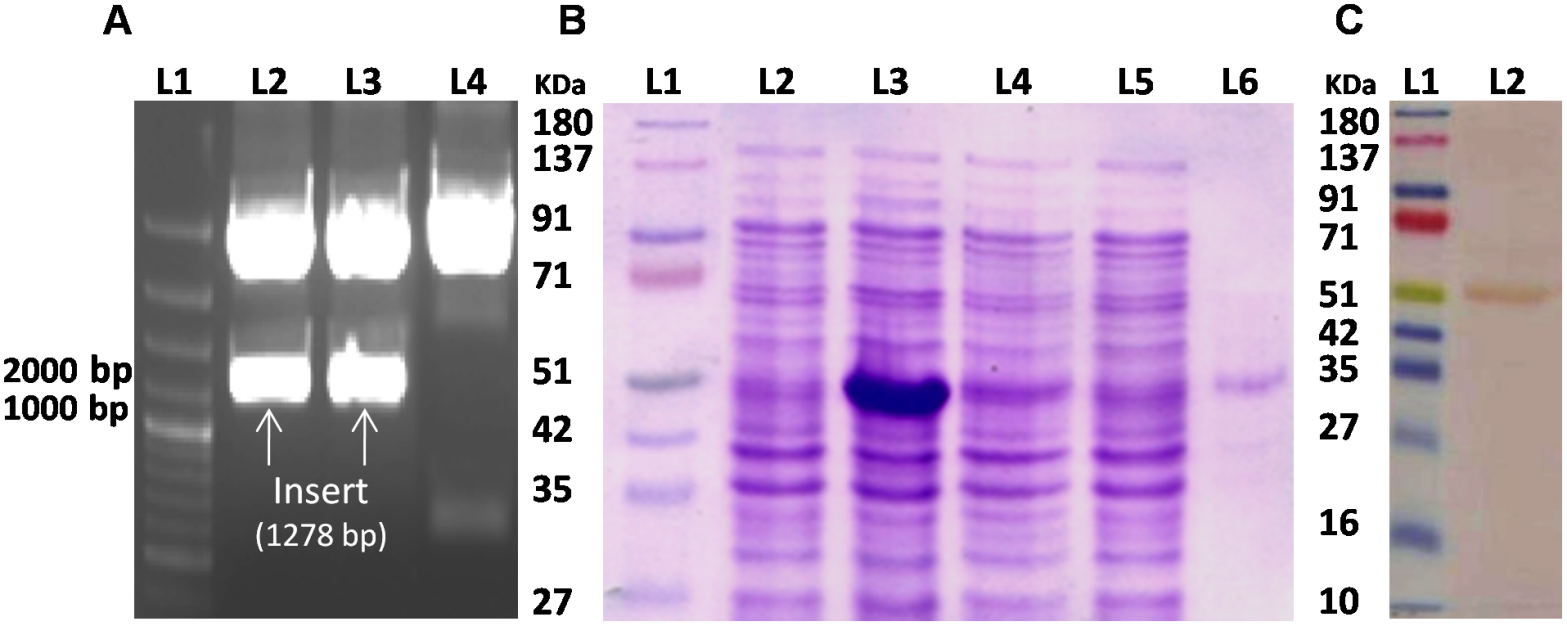
Confirmation of cloned construct, expression, purification and Western blotting of recombinant *w*Bm-MurA. **A:** The cloned gene within the expression vector pET28a was checked by restriction-digestion. Lane 1, molecular size marker (GeneRuler 1 kb Plus DNA Ladder, Thermo Scientific); lane 1–2, Restricted plasmid (insert-1278 bp); lane 3, un-restricted construct (pET28a containing the insert). **B:** Coomassie-stained SDS-polyacrylamide gel of recombinant *w*Bm-MurA over-expressed in Rosetta(DE3)pLysS *E. coli* strain with a His tag fusion protein. Lane 1, molecular mass markers (Puregene 4 Color Prestain protein ladder, Genetix); lane 2, uninduced *E. coli* lysate; lane 3; *E. coli* lysate after 22 h induction with 0.2 mM IPTG at 24°C; lane 4, flowthrough after passing the supernatant through an Ni-NTA column; lane 5, 10 column volumes eluted with wash buffer containing 60 mM imidazole; lane 6, purified *w*Bm-MurA recombinant fusion protein eluted with wash buffer containing 300 mM imidazole. **C:** Western blot developed with diaminobenzidine using mouse anti-His monoclonal antibody as primary antibody and HRP-conjugated anti-mouse IgG (lane 2) as secondary antibody; lane 1, molecular mass markers (Puregene 4 Color Prestain protein ladder, Genetix).

The *w*Bm-MurA gene of *Wolbachia* of *B. malayi* was successfully amplified and cloned in T/A vector. It was further sub-cloned in bacterial expression vector pET28a which was transformed into *E. coli* Rosetta strain. The recombinant construct was confirmed by restriction digestion with respective enzymes (Figure 4A) and also sequenced and it did not show any alteration in the amplified product. The soluble form of recombinant *w*Bm-MurA was expressed with fused (His)- tag at 24°C after inducing with 0.2 mM IPTG for 22 h at 24°C (Figure 4B). Extraction and purification yielded 0.3 mg of *w*Bm-MurA per liter of culture. The recombinant protein was localized with anti-His antibody through Western blot at ∼51 kDa (Figure 4C).

### *w*Bm-MurA is Present in the Various Life-stages of *B. malayi*

**Figure 5:**
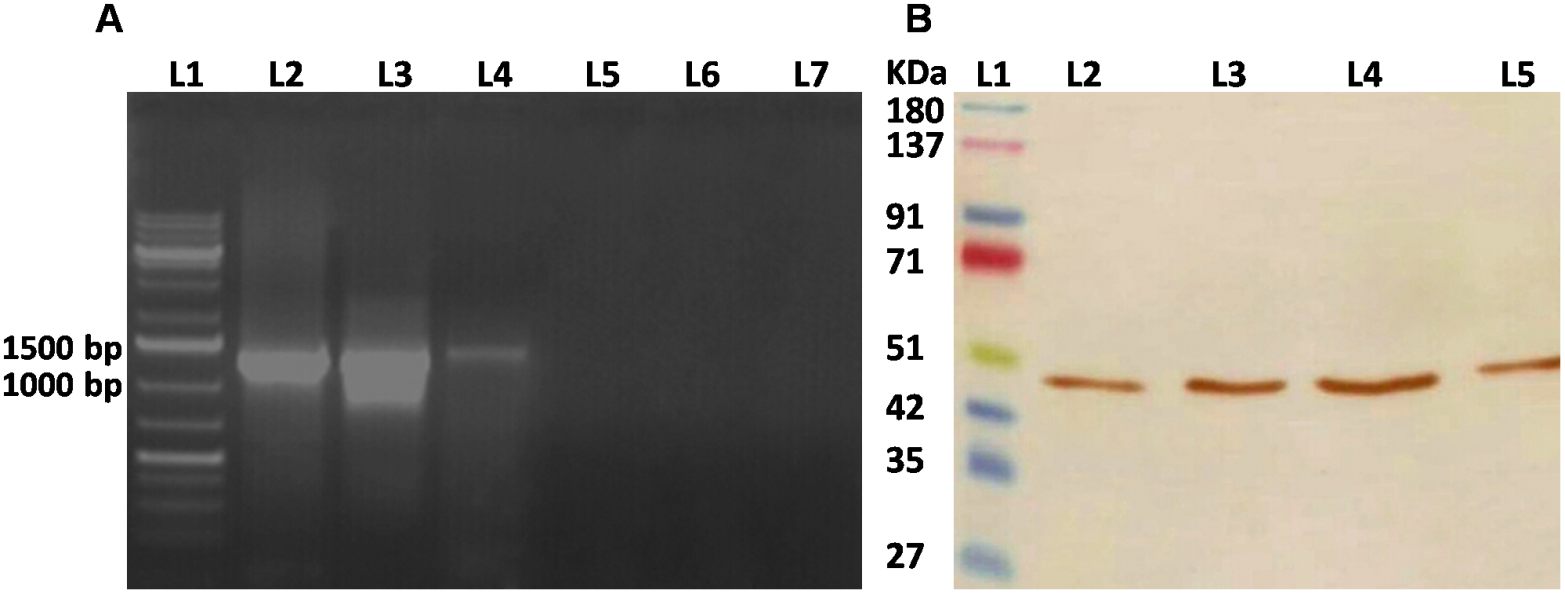
Stage specific expression of *w*Bm-MurA gene and the enzyme. **A:** Expression of *w*Bm-MurA gene. The full-length DNA (1278 bp, *w*Bm-MurA gene) was amplified from the cDNA of three life- stages of *B. malayi* using gene specific primers. Lane 1, molecular size markers (GeneRuler 1 kb DNA Ladder, Thermo Scientific); lane 2, infective larvae; lane 3, adults (both sexes); lane 4, microfilariae. Lane 5, 6 and 7 are controls containing PCR products from infective larvae, adults and microfilariae respectively in absence of reverse transcriptase. **B:** Endogenous protein (*w*Bm-MurA) expression. Western blot was performed with anti-*w*Bm-MurA antibody to confirm the presence of *w*Bm-MurA. Lane 1, molecular mass markers (Puregene 4 Color Prestain protein ladder, Genetix); lane 2, microfilariae; lane 3, infective larvae; lane 4, adult worms (both sexes); and lane 5, purified *w*Bm-MurA protein (positive control).

As shown in Figure 5A, the *w*Bm-MurA gene was found to be expressed in all the major life-stages of *B. malayi* which can be related to the presence of *Wolbachia* in all these stages. The polyclonal antibodies raised against the recombinant *w*Bm-MurA protein reacted in the blot with *w*Bm-MurA present in the crude extracts prepared from adults, L3 and mf. A characteristic band was visualized at ∼47 kDa while the purified *w*Bm-MurA taken as a positive control protein revealed ∼51 kDa band (Figure 5B). This marginal shifting in recombinant *w*Bm-MurA protein band could be either due to presence of His-tag, or some processing of MurA *in vivo,* or the presence of unintentional extra residues at N and C terminus of the recombinant protein during cloning.

### Confocal Immune Localization Revealed the Endogenous Presence of *w*Bm-MurA in Adult *B. malayi* and mf

**Figure 6:**
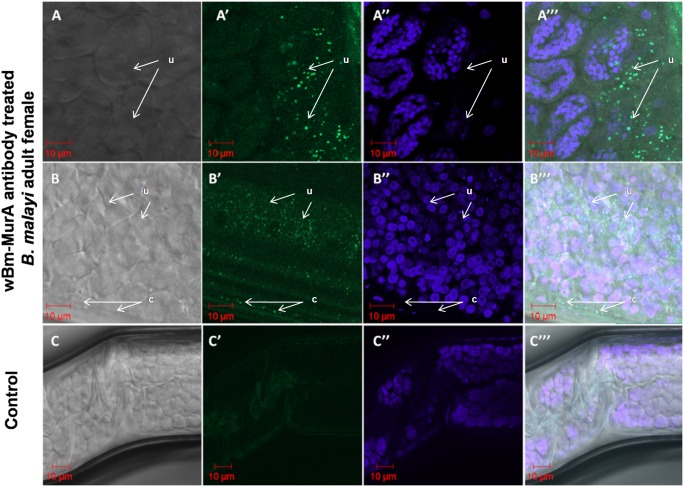
Immunolocalization of *w*Bm-MurA in female *B. malayi* adult worm by confocal microscopy. **A–A’’’ & B–B’’’:** Adult female worm was incubated with anti-*w*Bm-MurA polyclonal antibody followed by re-incubation with secondary FITC-IgG antibody and counterstaining with DAPI. **C–C’’’:** Adult female worm incubated with pre-immune serum followed by incubation with secondary FITC-IgG antibody and counterstaining with DAPI (control). A, B and C images are in Phase contrast; A’ and B’ demonstrate green fluorescence signal (dots) generated by FITC confirming the presence of *w*Bm-MurA; C’ has no green fluorescence signal in absence of specific antibody; A’’, B’’, C’’ show blue signals produced by DAPI indicating the presence of nuclear DNA; A’’’, B’’’, C’’’ are the merged images of phase contrast and the fluorescence. All the images were captured at 63X oil objective. u, uteri and c, lateral chord.

**Figure 7:**
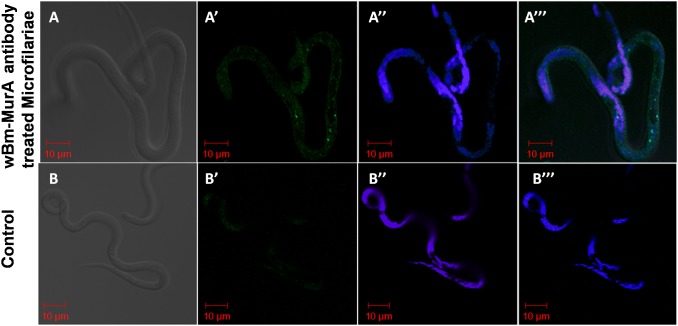
Immunolocalization of *w*Bm-MurA in microfilariae of *B. malayi* by confocal microscopy. **A–A’’’:** Microfilariae were incubated with anti-*w*Bm-MurA polyclonal antibody followed by re-incubation with secondary FITC-IgG antibody and counterstaining with DAPI. **B–B’’’:** Microfilariae were incubated with pre-immune serum followed by incubation with secondary FITC-IgG antibody and counterstaining with DAPI (control). A, B images are in Phase contrast; A’ demonstrate green fluorescence signal (dots) generated by FITC confirming the presence of *w*Bm-MurA; B’ has no green fluorescence signal in absence of specific antibody; A’’, B’’ show blue signals produced by DAPI indicating the presence of nuclear DNA; A’’’, B’’’ are the merged images of phase contrast and the fluorescence. All the images were captured at 63X oil objective.

The polyclonal antibody raised against *w*Bm-MurA in BALB/c mice reacted with *w*Bm-MurA protein within the adult female worm (Figure 6). Green fluorescence signal generated by binding of fluorescein isothiocyanate (FITC) tagged secondary antibody indicated the presence of *w*Bm-MurA expressed in *Wolbachia* within the uteri of the adult female worm and hypodermal lateral chords. The adult treated with the pre-immune BALB/c serum did not reveal such signal. *Wolbachia* and *B. malayi* DNA showed blue staining with DAPI (4′,6′–diamidino-2-phenylindole). *w*Bm-MurA was also localized as intense green signals in the isolated mf while those incubated with the pre-immune serum did not show any fluorescence (Figure 7).

### Kinetics of *w*Bm-MurA

**Figure 8:**
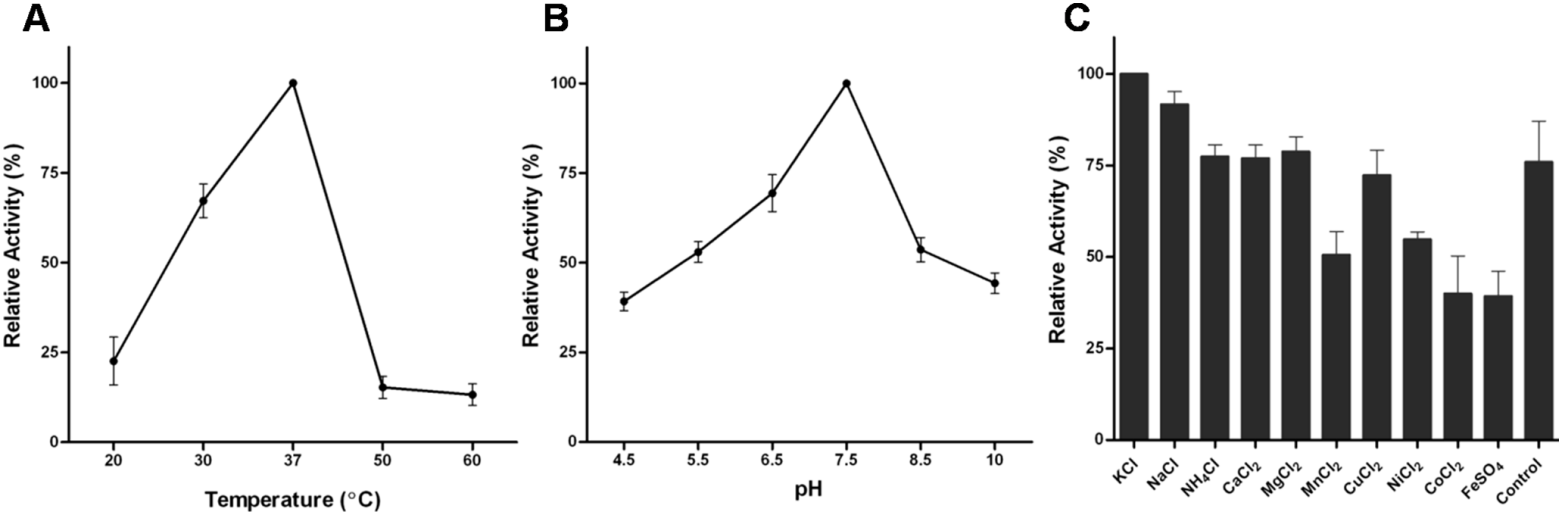
Effect of temperature, pH and ions on *w*Bm-MurA enzymatic activity. **A:** Temperature dependent enzyme activity profile assayed at various temperatures (20–60°C). **B:** pH dependent enzymatic activity at various pH (4.5–10). **C:** Effect of different ions (10 mM) on *w*Bm-MurA activity as compared to the control with no ions. Each Data point represents average of the three independent measurements. *Error bars* represent corresponding SEM.

**Figure 9:**
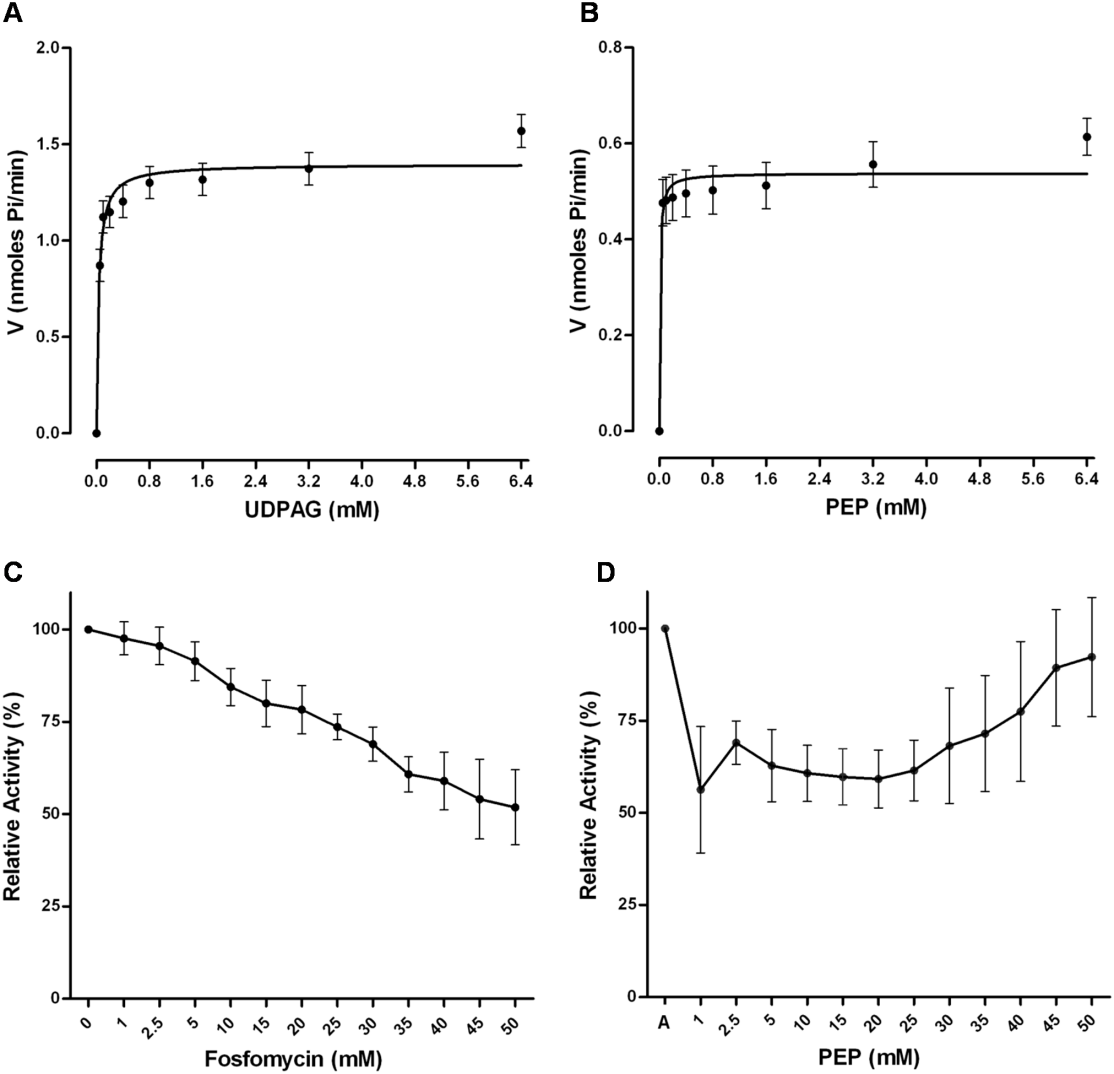
Kinetics profile of *w*Bm-MurA and inhibition of enzymatic activity by fosfomycin. **A:** The effect of UDPAG concentration on *w*Bm-MurA activity (*K*_m_: 0.03149 mM). **B:** The effect of PEP concentration on *w*Bm-MurA activity (*K*_m_: 0.009198 mM). *K*_m_ values were calculated by using the Michaelis-Menton plot. **C:** Relative effect of the fosfomycin on *w*Bm-MurA activity. **D:** The effect of PEP concentration on the inhibitory activity of fosfomycin, A value on X-axis is without fosfomycin. Each Data point represents an average of the three independent measurements. *Error bars* represent corresponding SEM.

The optimal pH and temperature were found to be 7.5 and 37°C respectively for enzymatic reaction (Figure 8A & B). The reaction mixture containing *w*Bm-MurA exhibited the release of inorganic phosphate unlike controls where the recombinant enzyme was either absent or was heat inactivated (Figure S1). The enzyme activity was inhibited in presence of manganese, copper, cobalt, ferrous and zinc ions (Figure 8C). Other ions have negligible effect on the activity as compared to potassium which was found to promote activity just like sodium. Based on initial velocity and the optimal conditions, the steady state kinetics was directly calculated. The *K*_m_ values for substrates were: UDPAG, 0.03149 mM; PEP, 0.009198 mM and the V*_max_* value for UDPAG and PEP were calculated as 1.397 mM/min/mg and 0.5378 mM/min/mg respectively (Figure 9A & B).

### Inhibitory Effect of Fosfomycin

Fosfomycin inactivates *w*Bm-MurA enzyme activity in a concentration dependent manner (Figure 9C). The enzymatic activity was found to be decreased by almost 2 fold at 50 mM concentration of fosfomycin at a fixed concentration of PEP. As the concentration of PEP increased, the enzymatic activity also increased (Figure 9D). This suggests that fosfomycin is a competitive inhibitor for *w*Bm-MurA as its inhibitory effect can be overcome by increasing the concentration of PEP.

### Homology Modeling

**Figure 10:**
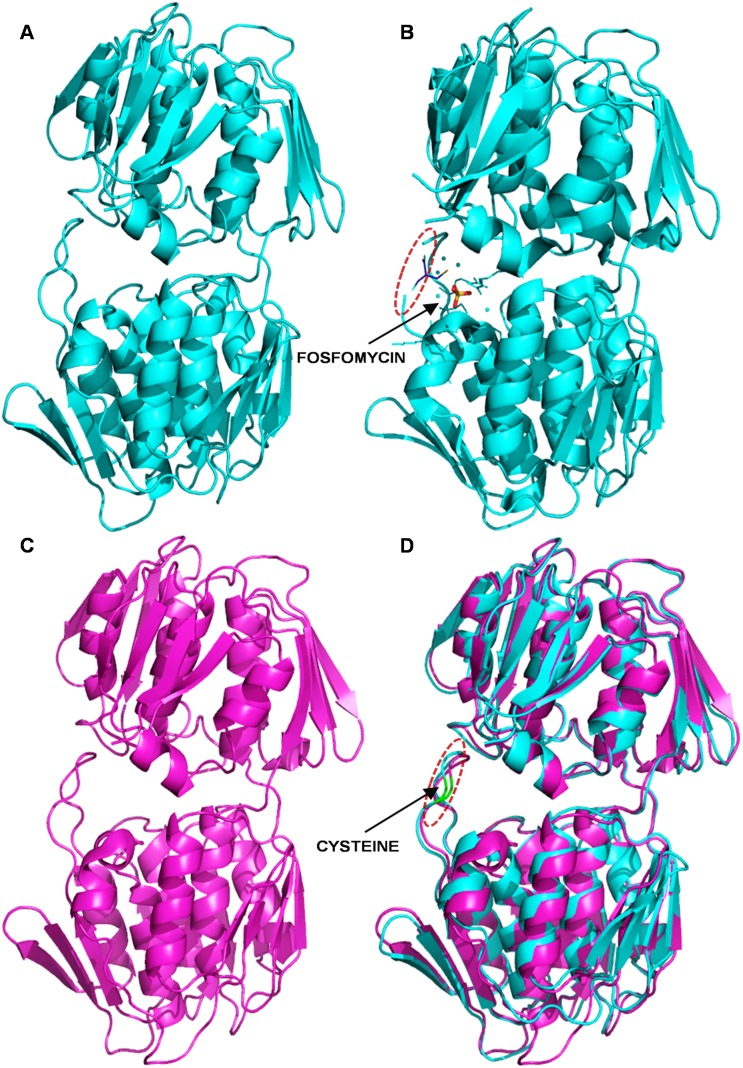
The homology modeling. **A (cyan):** Shows the simple unliganded structural model of MurA of *H. influenzae* (*Hi*-MurA, PDB: 2RL1). **B:** Technical view of the fosfomycin interactions during inhibition of the *Hi*-MurA. **C (magenta):** 3D refined homology structure model of *w*Bm-MurA. **D:** Superimposition of the *w*Bm-MurA homology model with the structure model of *Hi-*MurA. Green stretch (Cys124) in the loop of *w*Bm-MurA model fits with a similar green stretch (Cys115) of *Hi*-MurA which is the active site for fosfomycin.

The three dimensional model (Figure 10C) was 100% simulated at >90% confidence by using three different bacterial MurA as structure templates *viz*., *E. coli* (PDB: 1UAE) [44], *H. influenzae* (PDB: 2RL1) [45] and *Enterobacter cloacae* (PDB: 1EJD) [46] selected by sequence similarity with *w*Bm-MurA. The Ramachandran plot for a refined *w*Bm-MurA homology model revealed that 91.9% residues were in the most favored region, 5.5% in the additional allowed region, 2.1% in the generously allowed region and 0.5% in the disallowed region (Figure S2A), thus making the model geometrically acceptable. The z-score of *w*Bm-MurA homology modelfitted well within the range of experimentally determined similar X-ray solved protein structures (Figure S2B). The secondary structure analysis provides details that can be used to determine their topological features alongside the existing MurA solved structures. The final model consisted of 18 helices, 6 sheets, 24 strands, 5 beta hairpins, 1 beta bulge, 4 beta alpha beta motifs, 37 helix-helix interactions, 22 beta turns and 2 gamma turns which were consistent with the known MurA structures (Figure S2C). The model also has a surface loop containing the active site Cys124 at the specific region which was predicted to interact with the fosfomycin during enzyme inactivation. An overlay of our *w*Bm-MurA modeled complex with the MurA of *H. influenzae* template liganded with fosfomycin (Figure 10B) reveal proper superimposition of the active site in both the structures (Figure 10D). It is therefore suggested that the inhibitor (fosfomycin) would interact with the Cys124 of *w*Bm-MurA as in case of MurA of *H. influenzae.*

## Discussion

*Wobachia* was first identified in 1924 [47] however, it created enormous interest among scientific community in recent years. Up to 76% of the insect species along with other invertebrates including nematodes are reported to be infected with *Wolbachia* [48], [49]. In filarial nematodes, *Wolbachia* exerts control over the host viability and fecundity [50], [51]. Moreover, their role in the development of filarial pathology as a result of immune responses generated against the endosymbiont and/or their released products has also been established [52], [53]. Fully annotated genome of *Wolbachi*a of *B. malayi* and other known *Wolbachia* provides opportunities for delineating functional pathways involved in host-parasite interaction. Peptidoglycan (PG) biosynthesis is one of the pathways within the bacteria which are essential for both cell division and cell wall elongation processes. Till date, only two reports are available on the functional attributes of the enzymes involved in PG synthesis in *Wolbachia* [18], [54]. In the current investigation, characterization of one of such enzyme i.e. UDP-N-acetylglucosamine enolpyruvyl transferase of *B. malayi Wolbachia* (*w*Bm-MurA) has been undertaken which is evolutionary conserved throughout the bacterial species. The gene sequence of *w*Bm-MurA was successfully cloned, over-expressed and purified. We faced initial difficulty in purifying this His-tagged recombinant enzyme in soluble form since majority of the expressed protein was present in the inclusion bodies while soluble part contained very small amount of the protein. The protein present in the soluble part did not bind to Ni-NTA beads. Several attempts were made to bring the protein in soluble fraction such as low temperature, low IPTG concentration, purification buffers and variable pH which resulted in to marginal increase in its solubility. The recombinant enzyme was functionally active and appeared as ∼51 kD protein. The gene expression and the protein immuno-blotting studies indicated the presence of *w*Bm-MurA in adult worm, microfilariae and infective larvae of *B. malayi*. Localization of *w*Bm-MurA by confocal microscopy using anti-*w*Bm-MurA antibody further demonstrated its presence in the *Wolbachia* within the hypodermal chord and inside the uteri of female parasite i.e. in the developing embryos and mf. All this indicates that the enzyme is abundant during various stages of development of *B. malayi*. *w*Bm-MurA contains a single active EPSP synthase (pfam) domain that catalyses the chemical reaction with the release of inorganic phosphate. The optimal activity assay conditions such as pH and temperature defined the catalytic profile of the enzyme and the optimal enzymatic reaction occurred at pH 7.5. Interestingly, the cytoplasmic pH in *E. coli* is also maintained at pH 7.5 which may possibly reflect the activity of this enzyme at similar pH [55]. *w*Bm-MurA was active at various temperatures with optimal activity at 37°C. Several ions did not exhibit any significant effect on the enzyme activity except potassium which markedly enhanced it. The *K*_m_ value of *w*Bm-MurA for the substrate UDPAG (0.03149 mM) was comparatively lower than that of other bacterial MurAs (*E. cloacae,* 0.080 mM; *S. pneumoniae,* 0.244 mM; *S. mutans,* 0.12 mM and *S. aureus,* 0.168 mM) with the only exception being *E. coli* (0.015 mM) [56], [57]. For PEP (0.009198 mM) the value was higher than that of *E. coli* (0.0004 mM) and *E. cloacae* (0.0083 mM), while lower than that of *S. mutans* (0.086 mM) and *S. pneumoniae* (0.037 mM) [56], [57]. On the basis of these *K*_m_ values, *w*Bm-MurA appears to have lower affinity for both the substrates (UDPAG & PEP) as compared to that of *E. coli* MurA. It is difficult to explain the inferior substrate affinity in the current investigation may be attributed to the intracellular adaptation of symbiotic *Wolbachia* in *B. malayi*. Fosfomycin, a broad spectrum antibiotic irreversibly inhibits MurA. It acts as a PEP analogue and binds to MurA [21] leading to bacterial lysis and death [58]. *w*Bm-MurA enzymatic activity was demonstrated to be reduced in the presence of fosfomycin, however, PEP at increasing concentrations limits this inhibitory effect suggesting that fosfomycin competes with the PEP for its activity. Sequence and alignment analysis of *w*Bm-MurA with other bacterial MurA shows conservation of five important amino acid residues viz. Lys22, which participates in the formation of covalent adducts with PEP and fosfomycin [59], Cys115 and Asp305, which are involved in the product release and the final deprotonation step [60], Asp369 and Leu370 which facilitate interaction of fosfomycin with MurA [42] (all a.a. residues with ref. to *E. coli*). In *E. coli*, *M. tuberculosis* and *C. trachomatis,* it has been reported that a single change in the active site (cysteine by aspartate) makes MurA resistant to fosfomycin [43], [61]. Further, there is no replacement of active cysteine residue in *w*Bm-MurA demonstrating the nature of inhibition characteristic of fosfomycin. The conservation of these residues in the sequence further encourages us to investigate the consequences of differences in the active sites within *w*Bm-MurA structure. Thus a homology model based on three different well characterized bacterial MurA templates was developed. The *w*Bm-MurA structure is likely to contain 2 domains connected via double stranded linker containing fosfomycin active site. Superimposition of the *w*Bm-MurA model with that of *H. influenzae* demonstrated structural similarity in the fosfomycin binding site. Needless to say that residues belonging to active moieties in *w*Bm-MurA enzyme are well conserved.

As inferred from the annotated genome of *Wolbachia*, the full machinery of the PG biosynthesis is yet to be investigated. It has been suggested that *Wolbachia* has to retain lipid II biosynthesis setup in order to maintain cell division process [54]. Being intracellular, the fully polymerized cell wall of *Wolbachia* appears to be impractical and unusable [54]. MurA enzyme of the lipid II metabolism pathway is considered to be a validated antibacterial drug target. Several MurA inhibitors have been evaluated *in vitro* against bacterial MurAs and have exhibited excellent enzyme inhibitory activities. However, none was found very useful *in vivo* [62], [63]. The possible reason could have been their inability to cross the cytoplasmic bacterial membranes. In that case, drug delivery system would be an excellent method to target intracellular *Wolbachia*. Design and chemical synthesis of analogues of MurA inhibitors should be taken up to speed up the antifilarial drug discovery process and specific targeting of compounds to the filarial parasites within the lymphatic system.

To conclude the *w*Bm-MurA enzyme does not appear to be very different from other bacterial MurA and possesses quite similar sequence statistics along with active site residues and kinetic profile. Since this enzyme is constitutively expressed within the endosymbiont in all the major life stages we investigated and *Wolbachia* is required for worm survival and growth, *w*Bm-MurA may serve as a putative antifilarial drug target. The design and chemical synthesis of new molecules based on the available structure of MurA inhibitors is warranted to discover new adulticidal antifilarial agent.

## Supporting Information

Figure S1 **Malachite Green Assay of recombinant** ***w*** **Bm-MurA at 37°C.** The enzymatic reaction mixture with boiled *w*Bm-MurA (A), without *w*Bm-MurA (B) and (C) with purified recombinant *w*Bm-MurA. Each Data point represents average of the five independent measurements. *Error bars* represent corresponding SEM.
(TIF)

Figure S2 **Quality and assessment of the** ***w*** **Bm-MurA homology model. A:** Ramachandran plot from the PROCHECK server revealed the acceptable geometry of the *w*Bm-MurA homology model. **B:** The z-score of *w*Bm-MurA homology model. The score (−10.67) generated through ProSA-web server is within the range of experimentally similar X-ray solved MurA protein structures. **C:** Diagrammatic representation of the secondary structural elements of *w*Bm-MurA.
(TIF)
