# Supplementary figures and images for: Cloning, Expression and Characterization of UDP-N-Acetylglucosamine Enolpyruvyl Transferase (MurA) from Wolbachia Endosymbiont of Human Lymphatic Filarial Parasite Brugia malayi
Source: PLoS One. 2014 Jun 18;9(6):e99884. doi: 10.1371/journal.pone.0099884 (PMC4062475; doi:10.1371/journal.pone.0099884)

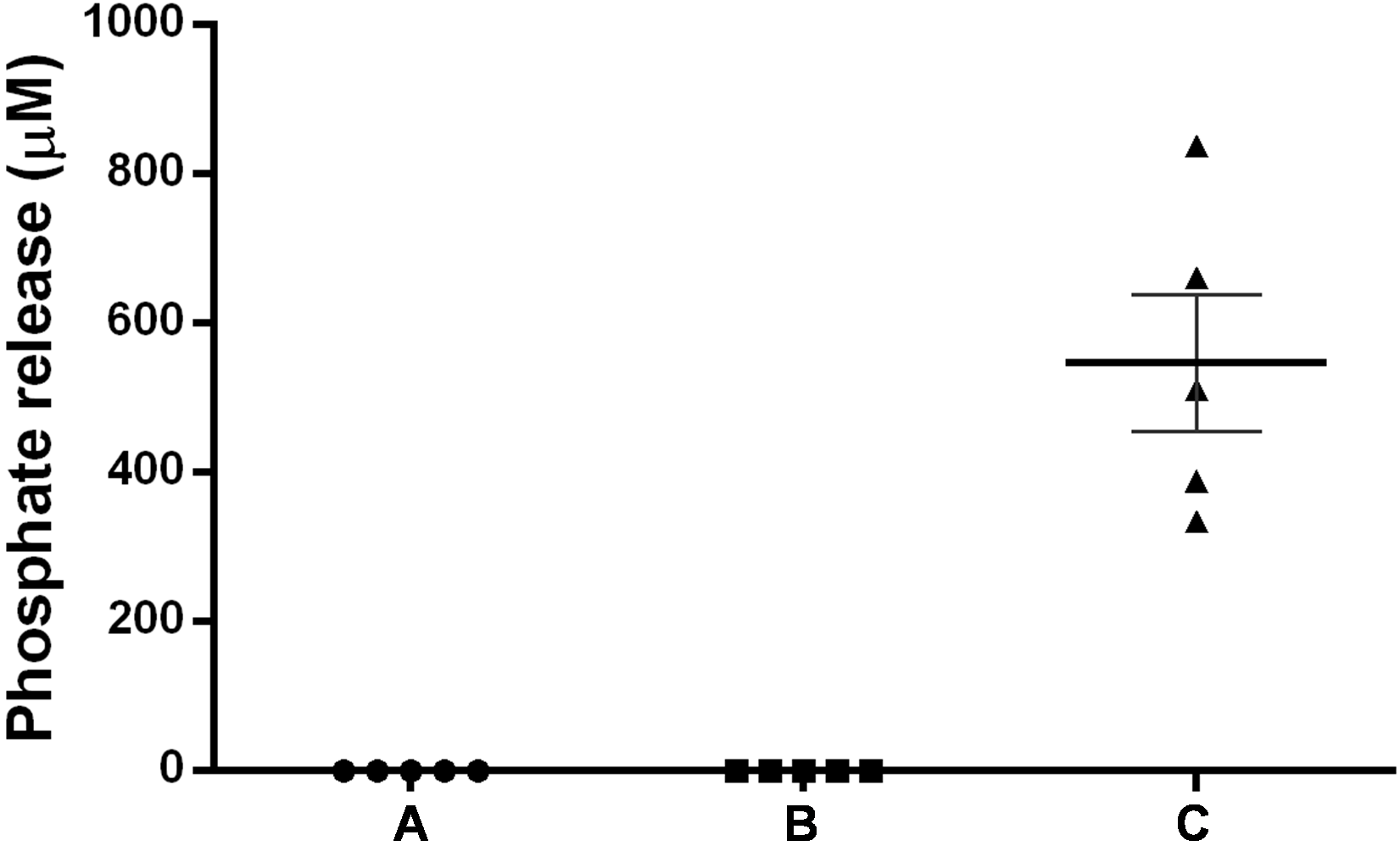

Supplement: Figure S1 — Malachite Green Assay of recombinant w Bm-MurA at 37°C. The enzymatic reaction mixture with boiled wBm-MurA (A), without wBm-MurA (B) and (C) with purified recombinant wBm-MurA. Each Data point represents average of the five independent measurements. Error bars represent corresponding SEM. (TIF) [file pone.0099884.s001.tif]

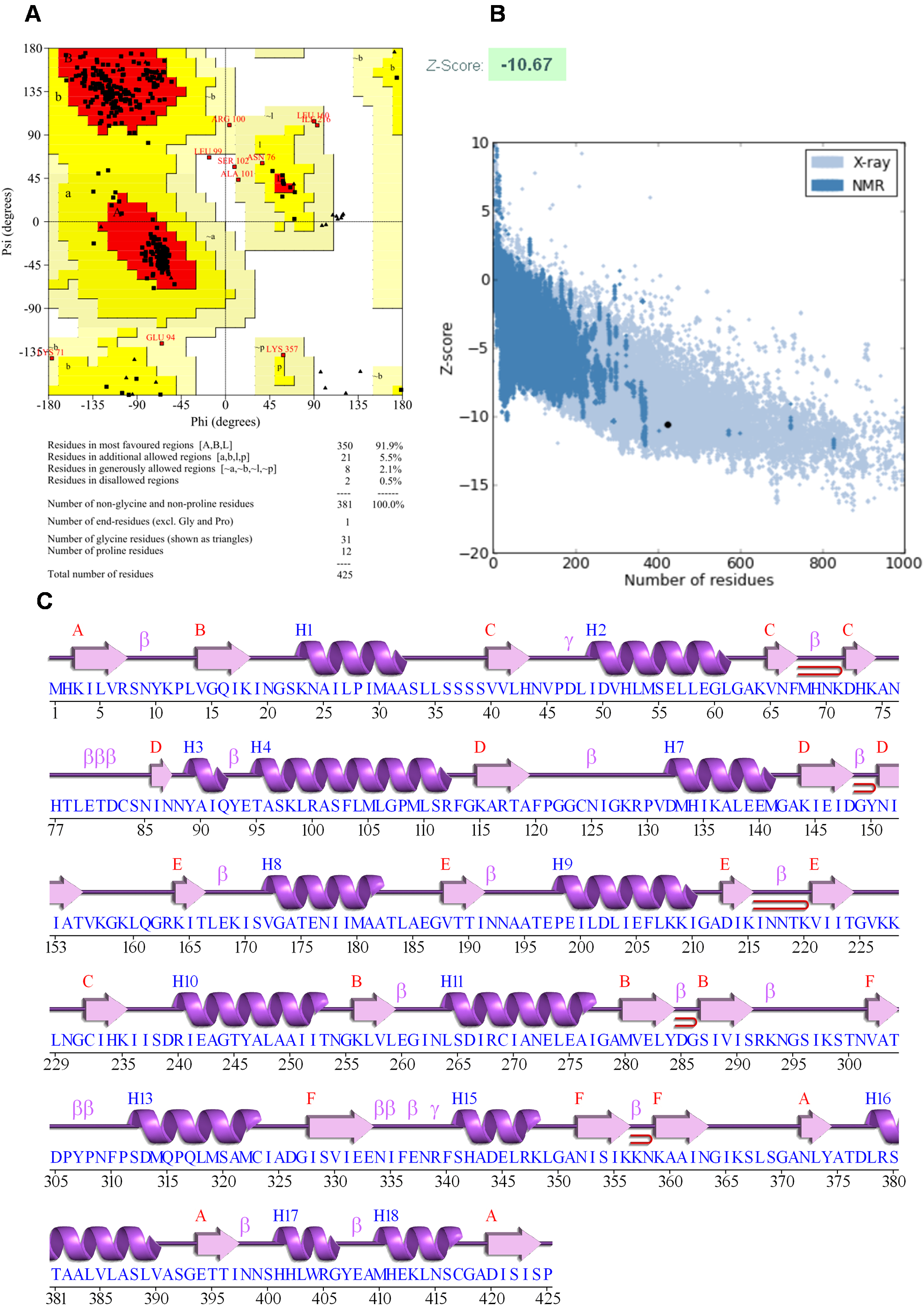

Supplement: Figure S2 — Quality and assessment of the w Bm-MurA homology model. A: Ramachandran plot from the PROCHECK server revealed the acceptable geometry of the wBm-MurA homology model. B: The z-score of wBm-MurA homology model. The score (−10.67) generated through ProSA-web server is within the range of experimentally similar X-ray solved MurA protein structures. C: Diagrammatic representation of the secondary structural elements of wBm-MurA. (TIF) [file pone.0099884.s002.tif]
